# *Nocardia takedensis*: a newly recognized pathogen responsible for skin and soft tissue infections

**DOI:** 10.1186/s12941-020-00379-7

**Published:** 2020-08-20

**Authors:** Romain Lotte, Alicia Chevalier, Sabine Dantas, Nicolas Degand, Alice Gaudart, Pierre-Simon Rörhlich, Laurent Boyer, Veronica Rodriguez-Nava, Pascal Del Giudice, Raymond Ruimy

**Affiliations:** 1 Department of Bacteriology, Nice Academic Hospital, Nice, France; 2 Université Côte D’Azur, CHU de Nice, Nice, France; 3 Université Côte D’Azur, Inserm, C3M, Nice, France; 4 Research Group On Bacterial Opportunistic Pathogens and Environment UMR5557 Écologie Microbienne, French Observatory of Nocardiosis, Institute of Infectious Agents, Hospices Civils de Lyon, Université de Lyon 1, CNRS, VetAgro Sup, Lyon, France; 5 Department of Pediatric Hematology, Nice Academic Hospital, Nice, France; 6 Department of Dermatology and Infectious Diseases, Centre Hospitalier Intercommunal, Fréjus-Saint-Raphaël, Fréjus, France

## Abstract

*Nocardia takedensis* was first isolated in 2005, from soil in Japan. We report here two cases of lymphangitis in France (2012–2017) caused by *N. takedensis* both occurring after skin injury while gardening, which enabled its inoculation. The two patients were immunocompromised and successfully treated by an antimicrobial agent active on the isolated strain, trimethoprim-sulfamethoxazole and amoxicillin-clavulanic acid for patient one and patient two, respectively. Our study along with previous ones supports the idea of a newly recognized cutaneous opportunistic pathogen and reinforces the recommendation of using gloves during soil exposure for immunocompromised patients. Lastly, according to data found in the literature, we would recommend trimethoprim-sulfamethoxazole as an efficient empirical antibiotic therapy in case of cutaneous infection caused by *N. takedensis*.

*Nocardia takedensis* was first isolated from soil in 2005 [1]. The first isolation in human was reported in 2006 in Japan [2]. Since then, five cases of cutaneous Nocardiosis caused by *N. takedensis* have been described during a seven-year period (2010–2017): one in the US [3], one in Mexico [4], two in Asia [5, 6] and one in France [7] (Table 1). We report two cases of lymphangitis caused by *N. takedensis* both occurring after skin injury while gardening. The patients were both immunocompromised and living in Southeast France. We discuss the source of contamination, the global dissemination of this *Nocardia* species, and the measures to prevent infection.

**Table 1:** Main features of reported cases of human infections caused by *N. takedensis*

Authors	Sex/age (years)	Underlying conditions(s)	Immunosuppression (risk factors)	Type of infection	Treatment	Inoculation	Geographical origin of the strain	Methods of identification	Evolution
Watanabe et al. [2]	M/75	T-lymphoma	Yes (T-lymphoma)	NA	NA	NA	Japan	Sequencing 16S rRNA gene	NA
Betran et al. [13]	F/41	Diabetes Eosinophilic granuloma	Yes (corticosteroid, diabetes)	Lung colonization	NA	NA	Spain	Sequencing 16S rRNA gene	NA
Tan et al. [5]	F/68	Diabetes Chronic kidney disease	Yes (diabetes, chronic kidney disease)	Foot cellulitis	Trimethoprim-Sulfamethoxazole	Unknown	Taiwan	Sequencing 16S rRNA gene	Recovery
Kresch-Tronik et al. [4]	M/33	NA	NA	Thoracic mycetoma	Trimethoprim-Sulfamethoxazole (800/160 mg/b.i.d) and Dapsone (100 mg/day)—14 months	Local traumatic injury	Mexico	Sequencing 16S rRNA gene	Recovery
Chung et al. [3]	F/68	Follicular lymphoma Myelodysplastic syndrome	Yes (corticosteroid, immunosuppressive treatment with sirolimus)	Lymphangitis of the left forearm	Cefuroxime (500 mg/b.i.d)—7 days Amoxicillin-clavulanate (875 mg/b.i.d)—14 days Trimethoprim-Sulfamethoxazole (800/160 mg/b.i.d)—7 days	Abrasion on the left dorsal hand	USA	Sequencing 16S rRNA gene	Recovery
Coussement et al. [15]	NA	Solid organ transplant	Yes (solid organ transplant)	NA	NA	NA	France	Sequencing 16S rRNA and *hsp65* genes	NA
Geun Lee et al. [6]	F/87	Arterial hypertension	No	Erythematous and swollen plaque on her left forearm Pustules, abscesses, ulcers	Trimethoprim-Sulfamethoxazole (400/80 mg/b.i.d)—40 days	Abrasion on the left forearm	Korea	Sequencing 16S rRNA gene	Recovery
Benzaquen et al. [7]	M/76	Marginal zone lymphoma Diabetes Arterial hypertension	Yes (corticosteroid, immunosuppressive treatment with chloraminophene and rituximab, diabetes)	Chronic ulcerative suppurative and painful nodules on the right forearm	Rifampicin (600 mg/day)—3 months Clarithromycin (500 mg/b.i.d)—6 months Ethambutol (800 mg/day)—6 months	Skin trauma	France	Sequencing 16S rRNA gene	Recovery
Lebeaux et al. [14]	NA	NA	NA	NA	NA	NA	France	Sequencing 16S rRNA gene	NA
Patient 1, 2012 (our study)	M/49	Myeloma	Yes (corticosteroid, immunosuppressive treatment with tacrolimus and lenalidomid)	Skin abscess and ulcerated nodular lymphangitis on the left forearm	Trimethoprim-Sulfamethoxazole—1 month	Skin trauma when gardening	France	Sequencing 16S rRNA gene	Recovery
Patient 2, 2017 (our study)	M/82	Myasthenia Diabetes Chronic kidney disease Arterial hypertension	Yes (corticosteroid, immunosuppressive treatment with azathioprin, diabetes, chronic kidney disease)	Skin abscess and nodular lymphangitis on the right forearm	Amoxicillin-clavulanic acid (1 g/t.id)—6 days	Hand skin wound when gardening	France	Sequencing 16S rRNA gene	Recovery

*NA *not available

## Study

Our study was performed in Nice Teaching Hospital, in Southeast France, during 1999–2019. A total of 50 isolates of *Nocardia* species were collected from 36 patients. The major types of infections were cutaneous infections (58%), pulmonary infections (33%) and bacteremia (9%). During the study period, all the isolates were identified to the species level using 16S rDNA gene sequencing as previously described [8].

**Fig. 1:**
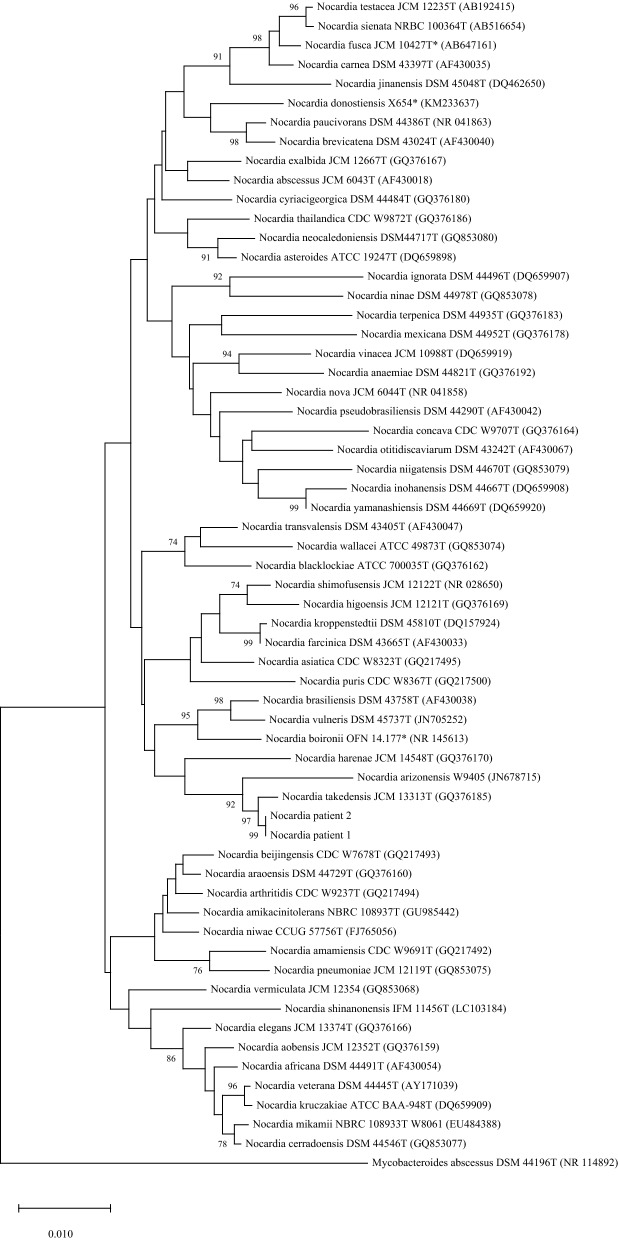
Phylogenetic tree based on the 16S rDNA sequence of *Nocardia* strains isolated from patients 1 and 2. Phylogenetic positions of the *Nocardia* strains isolated from patient 1 and patient 2 within the genus *Nocardia* based on the 16S rDNA sequences. The tree was obtained by Neighbor-joining method using MEGA X software [9]. Scale bar (= 0.01) indicates accumulated changes per nucleotide. The numbers next to the branches are the percentages determined in bootstrap analysis greater than 70% (1,000 replicates). The number in parentheses are the accession numbers of species in GenBank. *T* type strain,*species not yet validly published

For the two *N. takedensis* strains isolated from patient 1 and patient 2, blast analysis of the 1435 nucleotides amplicon both showed 99.6% identity to the 16S rDNA sequence of *N. takedensis* CIP108681^T^. Phylogenetic positions of the two clinical strains within the genus *Nocardia* are shown in Fig. 1. The tree was obtained by neighbor-joining method using MEGA X software as previously described [9]. The two isolates and *N. takedensis* CIP108681^T^ were compared using a random amplified polymorphic DNA-polymerase chain reaction (RAPD-PCR) with a coliphage M13 single stranded primer (5′-GAGGGTGGCGGCTCT-3). M13-PCR has been previously described by Guinebretière et al*.* for molecular typing of *Bacillus cereus* [10]. In the present work, this method was used to obtain and compare the RAPD-PCR profiles of *N. takedensis* isolates. Antibiotic susceptibility testing was performed using E-Test strips (bioMérieux) and CLSI interpretative standards for *Nocardia* spp.[11]. The two strains were sent to a French reference laboratory dedicated to *Nocardia* (French Observatory for Nocardiosis) to confirm AST results.

**Fig. 2:**
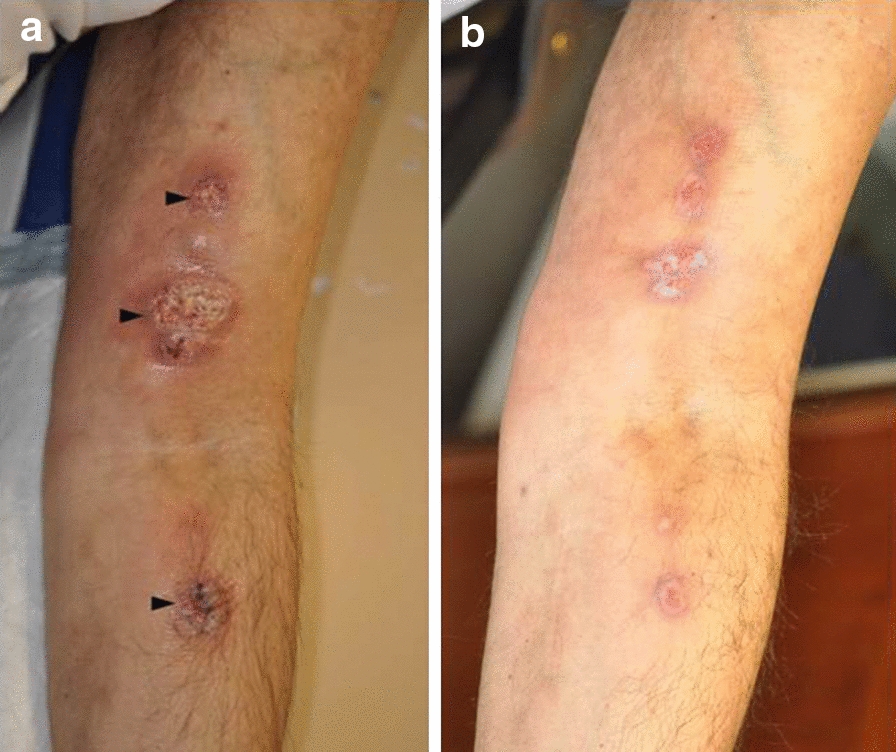
Ulcerated nodular lymphangitis of the left arm and forearm of patient 1. **a** Painful linear ulcerated nodules of the left arm and forearm (arrowhead) (on the first day of antibiotic treatment). **b** Healed lesions three weeks later

The first *N. takedensis* strain was isolated on November 19, 2012, from a 49 years-old male (patient 1) followed for a myeloma diagnosed in 2005. The patient had received allogeneic hematopoietic cell transplantation in 2008. In November 2012, he pricked his finger with a rose thorn while gardening at home without gloves in Fréjus (65 km from Nice). The patient attended the dermatology ward two weeks later, as a painful lesion had developed and spread from his hand to his left forearm. Physical examination by a dermatologist showed three inflammatory and painful ulcers distributed in a linear fashion, which suggested an ulcerated nodular lymphangitis with no regional adenopathy (Fig. 2). The body temperature was normal. The patient had an immunosuppressive therapy due to chronic GVHD (Graft-versus-host Disease) including prednisone 20 mg/day and tacrolimus, and for relapse of myeloma he received lenalidomid 15 mg/day and dexamethasone 40 mg/week. A bacterial (pyogenic bacteria, atypical mycobacteria, *Nocardia*) or fungal infection was suspected. Whole-body computed tomography (CT) scan was performed and a systemic invasive infection was ruled out. Biopsy samples of the lesions were sent for analysis and a presumptive antibiotic treatment by levofloxacin was initiated. Microscopic histological examination showed specific infection markers. Both Gram and Ziehl–Neelsen staining were negative. Chlorazol black fungal staining did not retrieve any mycelial filament. After 72 h, cultures on blood agar plates at 35 °C under 5% CO_2_ remained negative. The antibiotic treatment by levofloxacin was stopped after 3 days because of suspicion of Quinolone-induced Achilles tendinopathy. The dermatologist then switched the treatment to trimethoprim-sulfamethoxazole as he suspected a cutaneous Nocardiosis based on the first microbiological results (negativity of standard cultures after 72 h). Coletsos medium (Bio-Rad™, Marnes-la-Coquette, France) seeded for detection of atypical mycobacteria, grew *N. takedensis* under aerobic conditions at 30 °C in 4 days. The patient showed a rapid reduction of pain and cutaneous lesions within a week after the introduction of cotrimoxazole. He was considered cured after 1 month of treatment. Clinical and paraclinical long-term follow-up (every 2 weeks for multiple myeloma) excluded a late relapse of the nocardial infection.

The second *N. takedensis* strain was isolated on November 24, 2017, from an 82-years-old immunocompromised male patient (patient 2) treated with prednisone 15 mg/day and azathioprine 150 mg/day for myasthenia since 2016. He was hospitalized in November 2017 to manage an acute ocular myasthenia with altered general conditions. Physical examination revealed a painless and non-itchy cutaneous abscess measuring 6 mm in diameter on the palmar face of his right hand with serous and hematic fluid, as well as a nodular lymphangitis on his right forearm. The body temperature was normal. A thorough interview of the patient revealed that this lesion had developed a few days before his admission, following a minimal hand skin wound when gardening at home without gloves in Menton (30 km from Nice). Laboratory investigations revealed an elevated C-reactive protein (CRP) at 55 mg/L. Whole-body CT scan excluded a systemic infection. Before starting the antibiotic treatment, a sterile swab was used to collect pus from the abscess. Gram staining did not show any microorganism. Swab cultures grew *N. takedensis* after 72 h of incubation both on blood agar plate (Oxoid, Dardilly, France) under aerobic atmosphere and on chocolate agar plate under 5% CO_2_. The patient was discharged with a prescription of amoxicillin-clavulanic acid 1 g t-i-d for 7 days. The wound completely healed 10 days after the beginning of this treatment. Long term hospital follow-up of this myasthenic patient (every two months) excluded a late relapse of the cutaneous Nocardiosis.

Both *N. takedensis* isolates displayed the same antimicrobial susceptibility pattern: susceptible to beta-lactams, trimethoprim-sulfamethoxazole, linezolid, amikacin, tobramycin and minocycline but resistant to ciprofloxacin and clarithromycin (Table 2). To date, data concerning *N. takedensis* susceptibility to antibiotics are scarce. Only 8 isolates have been tested (including our study) and were susceptible to trimethoprim-sulfamethoxazole in vitro and 100% of the patients treated by an antibiotic treatment including trimethoprim-sulfamethoxazole completely recovered [5, 6, 12, 13]. Therefore, we assume that this molecule could be used as an empirical antibiotic therapy in case of cutaneous Nocardiosis due to *N. takedensis*, before an AST is available.

**Table 2:** Antimicrobial susceptibility testing determined using the E-test method

Antimicrobial agent	Strain patient 1		Strain patient 2	
	MIC (µg/ml)	Clinical categorization^a^	MIC (µg/ml)	Clinical categorization^a^
Amikacin	0.38	S	1	S
Amoxicillin-clavulanic acid	6	S	6	S
Ceftriaxone	1.5	S	0.25	S
Ciprofloxacin	32	R	2	I
Clarithromycin	3	R	4	R
Imipenem	0.25	S	0.125	S
Linezolid	1.5	S	0.19	S
Minocycline	1	S	0.125	S
Trimethoprim-sulfamethoxazole	0.047	S	1	S
Tobramycin	0.75	S	3	S

^a^Interpretations of MICs in terms of susceptibility for *Nocardia* according to the criteria of CLSI [11].

**Fig. 3:**
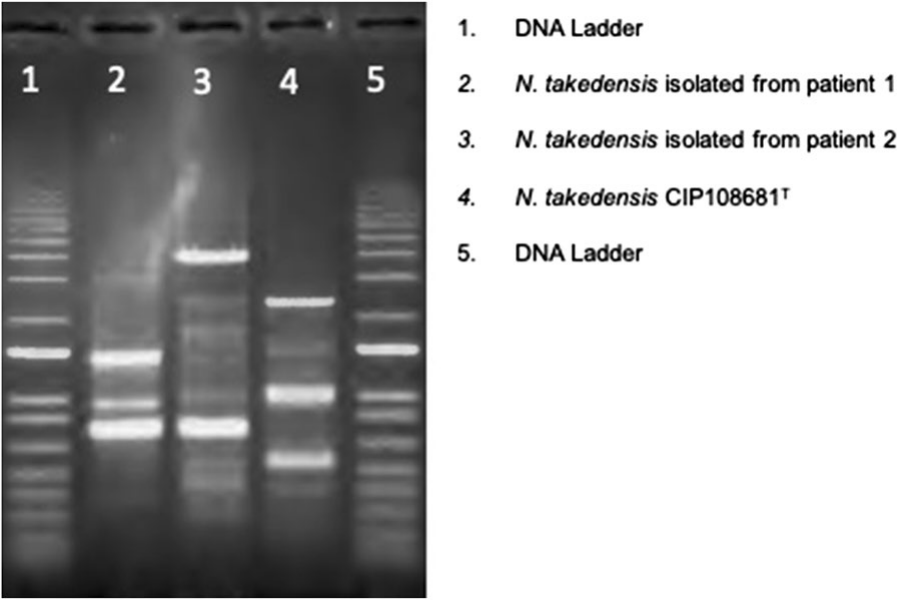
Molecular typing using M13-PCR methods, as described by Guinebretière et al*.* [10]. *Nocardia takedensis* strains isolated from the two patients and *Nocardia takedensis* CIP108681^T^

The M13-PCR typing revealed that the two patients were infected by 2 different strains and excluded a clonal transmission between the 2 patients or a same origin of the strains. The two strains isolated in Southeast France were also different from the type strain *N. takedensis* CIP108681^T^ isolated in Japan (Fig. 3). *N. takedensis* was first characterized in 2005 by Yamamura et al. [1]. The two first reference strains of *N. takedensis i.e.* MS1-3t and AS4-2 were isolated in Japan respectively from a moat sediment and from sludge obtained from a sewage treatment plant. To date, only nine clinical isolates of *N. takedensis* have been reported in human [2–7, 13–15]. The main clinical and microbiological features of *N. takedensis* isolates are summarized in the Table 1. Including the two patients of our study, the mean patients’ age was 64 years (33–87 years), with a sex ratio of 1.25. *N. takedensis* is mostly involved in primary cutaneous Nocardiosis, just as the closely related species *N. brasiliensis* is [16, 17]. Indeed, seven out of eight infected patients (88%) presented various forms of skin and soft tissue infections such as cellulitis, chronic skin ulceration, lymphangitis, or mycetoma, that occurred several days after a skin lesion allowing bacterial inoculation [3–7, 15, our study]. In one patient *N. takedensis* was isolated from a respiratory specimen but without any clinical or radiological signs of pulmonary Nocardiosis, therefore corresponding to a transient pulmonary colonization [13]. For the last three clinical isolates reported in the literature, no clear and substantial data referring to the clinical cases were available [2, 14, 15].

Collectively, these cases of primary cutaneous nocardiosis reported in US [3], Mexico [4], Asia [5, 6], and Europe [7, our study], during 2010–2017 support the idea of *N. takedensis* as a newly recognized pathogen involved in skin infections. An interesting hypothesis is that climatic and soil conditions could have play a role in the environmental spread of this bacterial species. Indeed, in most of the cases published in the literature, *N. takedensis* strains were isolated in geographical area with favorable climatic conditions (South–East France, Spain, Korea, Japon, Taiwan or Mexico) [2, 4–7, 13, our study]. Moreover, Brown-Elliott et al., have previously suggested that *Nocardia brasiliensis*, a species that is phylogenetically closely related to *N. takedensis*, is predominantly isolated in tropical or subtropical region [16]. Recently, Lebeaux et al., showed a higher density of *Nocardia* isolates in the South of France and overseas territories than in other French areas, suggesting that environmental conditions may also increase the spread of *Nocardia sp* [14]. To date, data found in the literature concerning the potential impact of the climatic conditions on soil and on *N. takedensis* spreading are scarce and further studies will be necessary to assess this challenging hypothesis.

Concerning the source of contamination, it is likely possible that the two patients got infected by traumatic inoculation of *N. takedensis* present in the soil as both of them were gardening without gloves in the days preceding the onset of the symptoms. Furthermore, the two patients had received immunosuppressive drugs and corticosteroids which could have played a role in the infection. Immunosuppressive therapy or known risk factors of immunosuppression were found in all but one patient with *N. takedensis* infections (Table 1).

## Conclusions

With the spread of 16S rDNA sequencing, new *Nocardia* species have been recently described and the genus *Nocardia* currently encompasses more than 50 species involved in human diseases [16–18]. Besides, the rising number of patients receiving immunosuppressive therapies increases the risk of Nocardial infection through environmental exposure. Our study emphasizes the idea of *N. takedensis* as a newly recognized opportunistic pathogen responsible for skin infections. Infections occur after an inoculation following a cutaneous lesion. As this bacterium is found in the environment especially in soil, physicians should recommend their immunocompromised patients to wear shoes as well as clothing covering the skin when they are working in the soil (https://www.cdc.gov/nocardiosis/prevention/index.html). More, we would strongly recommend to immunocompromised patients to wear gloves when manipulating soil. This recommendation could also be extended to non-immunocompromised patient as primary cutaneous Nocardiosis has been repeatedly reported in this population. Lastly, because of the slow growth of *Nocardia* sp., prolonged culture conditions are required when a cutaneous Nocardiosis is suspected.

## Data Availability

All datasets on which the conclusions of the manuscript rely are presented in the main paper and in the figures and tables.
